# Research progress and future prospects of antimicrobial modified polyetheretherketone (PEEK) for the treatment of bone infections

**DOI:** 10.3389/fbioe.2023.1244184

**Published:** 2023-08-03

**Authors:** Ziyi Zhang, Junxing Shao, Yu Gao, Yuhuan Li, Te Liu, Modi Yang

**Affiliations:** ^1^ Department of Orthopedics, China-Japan Union Hospital of Jilin University, Changchun, Jilin, China; ^2^ Scientific Research Center, China-Japan Union Hospital of Jilin University, Changchun, Jilin, China

**Keywords:** polyetheretherketone, antibacterial properties, modification, biomaterials, bone infection

## Abstract

Infection of the bone is a difficult problem in orthopedic diseases. The key and basis of the treatment of bone infection is the effective control of local infection, as well as the elimination of infection focus and dead cavities. The most commonly used approach utilized for the prevention and management of bone infection is the application of antibiotic bone cement. However, the incorporation of antibiotics into the cement matrix has been found to considerably compromise the mechanical characteristics of bone cement. Moreover, some investigations have indicated that the antibiotic release rate of antibiotic bone cement is relatively low. Polyetheretherketone (PEEK) and its composites have been considered to perfectly address the challenges above, according to its favorable biomechanical characteristics and diverse surface functionalizations. This article provides a comprehensive overview of the recent advancements in the antimicrobial modification of PEEK composites in the field of antibacterial therapy of bone infection. Furthermore, the potential application of PEEK-modified materials in clinical treatment was discussed and predicted.

## 1 Introduction

Infection of the bone has emerged as the primary cause of high mortality in orthopedic diseases (Nair et al., 2000; Thabit et al., 2019). In orthopedics, infections of bone tissue, such as suppurative arthritis, osteomyelitis, bone and joint tuberculosis, bone and joint syphilis, and prosthetic joint infection (PJI), continue to be a challenging issue. A large number of amputations and even deaths are reported annually as a result of bone-related infections (Ercan et al., 2011). *Staphylococcus aureus*, Methicillin-resistant *S. aureus* (MRSA), *Escherichia coli*, *Pseudomonas aeruginosa*, *Streptococcus,* and others are prevalent pathogens (Sarkissian et al., 2016; Kerneis et al., 2017; Lebowitz et al., 2017; Arciola et al., 2018). Bone infection can significantly impair local tissue and the regeneration ability of bones, making the defect difficult to heal, and leading to formation of dead bone and cavities. The critical step of bone infection treatment is powerful and effective infection control *in situ* and bone defect repair (Ansari, 2019; Afewerki et al., 2020). Only when the infection is controlled, the repair of the bone defect commences. Thus, Comprehensive removal of infection foci and the local release of antimicrobial drugs are especially crucial. Currently, intravenous antimicrobials and topical antibiotic extended-release systems are synergistically employed to control infection (Masters et al., 2022). In addition, the disease is difficult to treat and requires a lengthy therapeutic course, because antibiotic penetration into the infected site through the dead bone cavities or bacterial biofilm (BBF) on the internal fixators or protheses is required (Chen et al., 2021b). The primary mode of infection in bone is typically hematogenous, resulting from bacteremia, or through direct bone tissue penetration (Kurtz et al., 2008; Mathew and Ravindran, 2014). As a result, the administration of systemic intravenous antibiotics has proven to be an efficacious method for both prevention and treatment. Prolonged administration of systemic antibiotics may result in adverse effects such as hepatic and renal impairment, bacterial resistance, and in severe instances, fungal infections such as candidiasis, and so on. Antibiotics should be carefully selected for patients with low body weight and abnormal circulatory function (Barber et al., 2016). The use of an antibiotic sustained-release system can deliver higher concentrations of antibiotics to the site of infection, thereby reducing the incidence of systemic adverse reactions while effectively controlling the infection (Janz, et al., 2016; Anagnostakos and Fink, 2018; Thabit, et al., 2019; Buck, et al., 2020). Therefore, the development of orthopedic implants with remarkable sustained-release characteristic of antimicrobials and favorable biomechanics similar to bone offers a reasonable way for bone infection therapy (Hasan et al., 2013; Ouyang et al., 2016). As a result, antibacterial bone cement has become a widely utilized and the first choice for bone infection (Gandhi et al., 2018; Jameson et al., 2019; Yayac et al., 2019; Leong et al., 2020; Abdel Khalik et al., 2023).

More medical applications of bone cement are emerging as a consequence of the aging of the human body, which is accompanied by a gradual decrease in bone strength, and the rise in the number of accidents (Wekwejt et al., 2019). The inherent antibacterial efficacy of pure bone cement is limited, however, the incorporation of antibacterial metal ions and antibiotics during its production can confer antibacterial properties (Vaishya et al., 2013; Anderson et al., 2015; Radha et al., 2017). Polymethyl methacrylate is the most prevalent type of locally implanted bone cement (Valencia Zapata, et al., 2019; Gandomkarzadeh et al., 2020). Nevertheless, bone cement has been found to exhibit cytotoxic properties, leading to various adverse reactions such as impaired bone remodeling in the vicinity of the implant tissue, as well as local soft tissue fibrosis or tissue necrosis (Hoess et al., 2016; Inzana et al., 2016; Robo et al., 2018). It even leads to excessive activation or inactivation of platelets that affects hemostasis (Anderson et al., 2015), and even the appearance of bone cement implantation syndrome (Donaldson et al., 2009). In addition, the incorporation of antimicrobial agents into cement results in a decrease in its rigidity (Schiavone Panni, et al., 2016). To perform first-stage bone infection associated surgery and simultaneously eradicate adverse effects after bone cement implantation, PEEK and its modified materials have been widely employed by researchers as a novel alternative to bone cement for managing localized bone infections.

PEEK is a two-phase, semi-crystalline polymer with excellent flame retardancy, hydrolysis resistance, radiation resistance, and chemical and biological stability. PEEK’s elastic modulus is significantly lower than that of metal titanium (Ti), resembling that of bone tissue (Carpenter et al., 2018). Furthermore, PEEK is rigid, making it highly resistant to cyclic stress (Buck et al., 2020; Flejszar and Chmielarz, 2020) and stress shielding, thereby reducing the effect of bone lysis. Moreover, PEEK’s main advantage is its high plasticity, which allows it to be shaped into a variety of shapes using three-dimensional (3D) printing technology (Kurtz and Devine, 2007). Therefore, PEEK has been extensively utilized in orthopedic bone defect repair, implants, and joint replacement (Hamdan and Swallowe, 1996; Rae et al., 2007). Additionally, PEEK is a biologically inert substance. The hydrophobic nature of PEEK’s molecular structure renders it unsuitable for cell adhesion (Gu et al., 2020; Oladapo et al., 2021). The osseointegration capability of pure PEEK is limited. However, this characteristic confers a favorable attribute of reducing bacterial adhesion to the material’s surface. Much more research in this area is initial to focus on efforts to promote osseointegration and reduce bacterial colonization and reproduction—both of which are crucial to the long-term effectiveness of implants. In recent years, scholars have made significant contributions to the advancement of PEEK (Han et al., 2022). The modified PEEK material possesses the property of being injectable while maintaining the original rigidity of the material to a significant extent and exhibiting no apparent adverse reactions, thereby providing an advantage over bone cement. This paper presents classification and discussion of the modified materials of PEEK with anti-infection properties, as developed by researchers in recent years, through a comprehensive review of relevant literature.

## 2 The strategy of strengthening the antibacterial activity of modified materials

**FIGURE 1:**
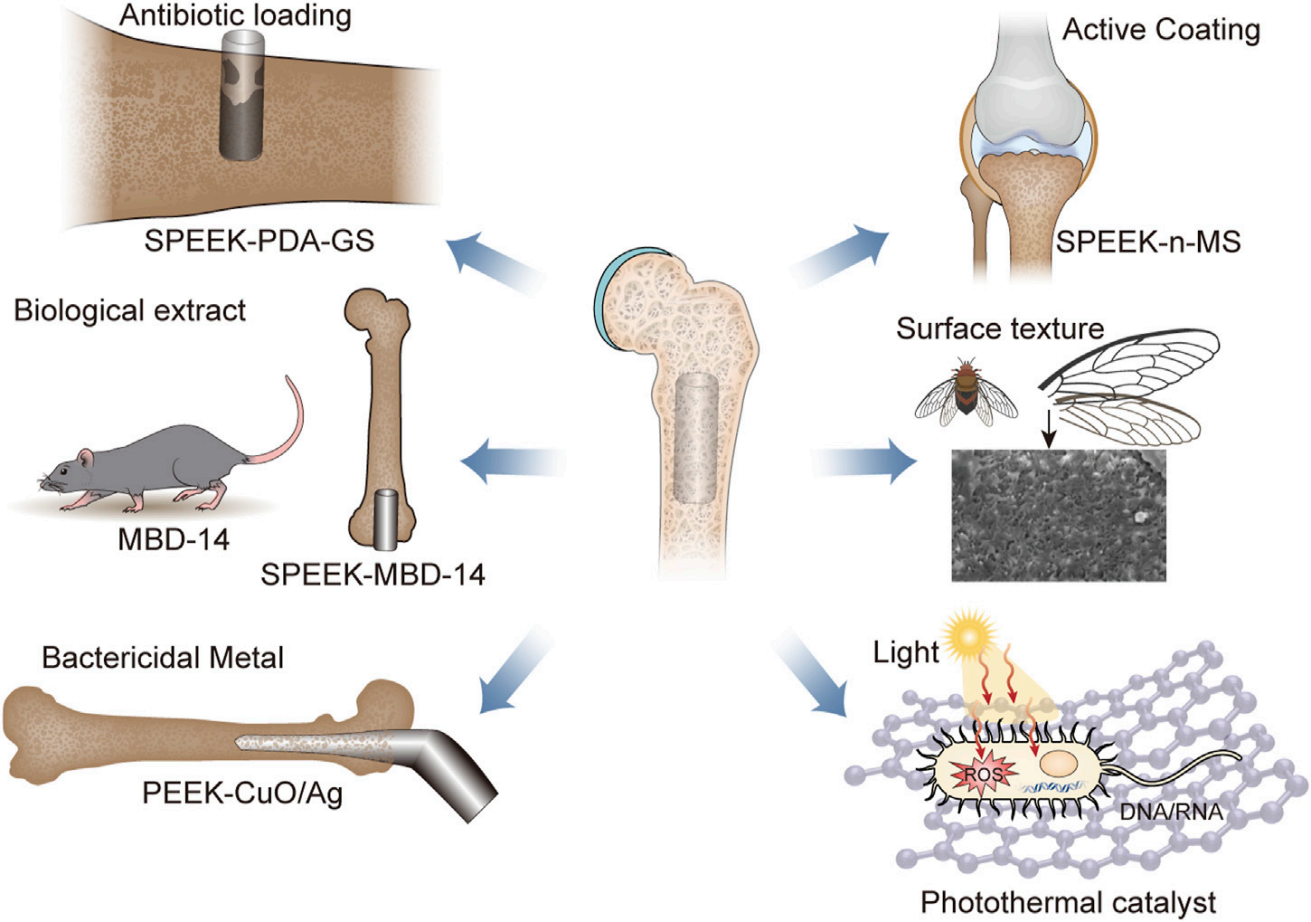
The main classification of antibacterial surface modification of PEEK, including load antibiotics, biological extract, bactericidal mental, active coating, surface texture, and photothermal catalyst. Abbreviation: SPEEK, sulfonated polyether ether ketone; GS, Gentamicin sulfate; MBD-14, mouse-defensin-14; n-MS, nano-magnesium silicate.

It was widely reported that poor osseointegration and bacterial infections had been identified as the primary factors contributing to the failure of PEEK and its composite implants (Koch et al., 2010; Khonsari et al., 2014). The bacteriostatic properties and osseointegration potential of PEEK and its composites were enhanced through the implementation of surface modification methodologies, including surface coarsening, chemical functionalization, and coating (Sun et al., 2018; Yu et al., 2020; Xie et al., 2021). As Figure 1 and Table 1 shown, the modified PEEK for the treatment of bone infections were briefly described.

**TABLE 1:** Antibacterial modification strategies on PEEK and its composites for antibacterial properties.

Modification strategy	Modification coatings	Results related to antibacterial property *in vitro* study	Results related to antibacterial property *in vivo* study	References
Loaded antibiotics	Van, ZnO, Amp	Had good inhibitory effect on the growth of *Staphylococcus aureus*	-	Chen et al. (2019)
	TOB, PDA, GelMA	Defeated osteosarcoma cells and bacteria	Excellent osteogenic activity	Yin et al. (2020)
	GS, CaP	Excellent and sustained antibacterial property, biocompatibility and cell osteogenic differentiation	Exhibited *in vivo* antibacterial activity and osseointegration ability in the treatment of bone defect with infection	Xue et al. (2020)
	GS, PDA	Impeded bacterial proliferation and exhibited anti-inflammatory characteristics	Excellent osteogenic activity and antimicrobial properties	Sun et al. (2021)
	Dex/Mino liposomes	Enhance the osseointegration and antibacterial efficacy of biomaterials	Enhanced osteointegration, and antibacterial activity in beagle femoral implant models	Xu et al. (2019)
	β-lactam antibiotics	Inhibited the reproduction and growth of *Staphylococcus aureus*	-	Montero et al. (2016)
AMPs	KR-12, PDA	improved antibacterial activity against *Staphylococcus aureus*	Increased osteointegration in rats, improved antibacterial activity against *Staphylococcus aureus*	Meng et al. (2020)
	MBD-14	Exhibited long-term antibacterial activity against gram-positive and gram-negative microorganisms	Stimulated osseointegration and protein expression	Yuan et al. (2019)
	GL13K, EDC	Provided bactericidal activity and biofilm resistance	—	Hu et al. (2021)
Loaded plant polyphenols	HK, nano-bioglass	Exhibited potent antibacterial activity against *Staphylococcus aureus*	Did not significantly promote bone formation	Zhang et al. (2018)
	Lawsone, bioactive glass	Exhibited potent antibacterial activity against *Staphylococcus aureus*	—	Ur Rehman et al. (2018)
	RV, nano-porous magnesium calcium silicate	Antimicrobial and osteogenesis activity	—	Karimi-Soflou et al. (2022)
	Genistein, nano-porous tantalum pentoxide, sulfuric acid	Inhibit the growth of *Escherichia coli* and *Staphylococcus aureus*	Increased bone integration, stimulated the response of BMSC	Mei et al. (2021)
	CGA, hydrogel	Suppressed *Escherichia coli* and *Staphylococcus aureus*	-	He et al. (2019)
	CR, GS	Prevents the reproduction of *Staphylococcus aureus* and *Escherichia coli*	Accelerated osteogenesis and osseointegration	Zou et al. (2020)
Loaded metal ions	Ag NPs, PDA	Reduced the reproduction of gram-positive and gram-negative bacteria	-	Deng et al. (2017)
	Ag NPs	Increased antibacterial activity	Improves adhesion of PEEK implants to bone	Liu et al. (2017)
	Ag NPs, GS, silk fibroin	Exhibited superior antibacterial properties	-	Yan et al. (2018a)
	Ag NPs, CMC, BFP	Exhibited an outstanding inhibitory effect on bacteria, promoted osteogenic differentiation and cell proliferation	-	Yu et al. (2021)
	Cu NPs	Effectively captured MRSA	Effectively captured MRSA	Liu et al. (2019)
	Cu nanoclusters, PDA, citrate	Enhanced the bacteria-killing ability	Encouraged bone regeneration of implants	Yan et al. (2021a)
	CuO, Ag NPs, silk fibroin, PDA	Presented synergistic antibacterial ability, potentiated osteodifferentiation	Increased bone integration, stimulated the response of BMSC	Yan et al. (2020)
	Mg	Demonstrated a significant bactericidal effect against *Staphylococcus aureus,* enhanced the biological activity	-	Yu et al. (2018)
	Ti/Mg/Ag gradient composite coatings	Improve the antibacterial ability, biological activity and bone conductivity	-	Gümüş et al. (2017)
	Nano-magnesium silicate	Exhibited notable antibacterial properties against *Escherichia coli* and *Staphylococcus aureus*, enhanced the differentiation of BMSCs	-	Niu et al. (2020)
Loaded photothermal catalyst	GO	Enhanced the antibacterial properties and biocompatibility of materials against *Escherichia coli,* promoted cell proliferation and differentiation	-	Ouyang et al. (2018a)
	GO nanosheets, PDA, oligopeptides	Impeded the proliferation of bacteria, lead to cellular disintegration	Enhanced cytocompatibility and promoted bone formation	Wang et al. (2020)
	GO, carbon fiber, Ti-6Al-4V	Exhibited a remarkable inhibitory effect on *Staphylococcus aureus*, enhanced cytocompatibility	-	Qin et al. (2021)
	GO, PDA, Dex- liposomes	Enhanced cellular adhesion and migration, as well as improve cellular biocompatibility	-	Ouyang et al. (2018b)
Doped bioactive coating	Hydrofluoric acid	Exhibited inhibitory effects on *Porphyromonas gingivalis*	Enhanced the osseointegration	Chen et al. (2017)
	N-FHA	Reduced bacterial expansion and biofilm development, exhibited enhanced cell adhesion and proliferation	Promoted osseointegration	Wang et al. (2014)
	Se NPs	Exhibited significant inhibition of *Pseudomonas aeruginosa*	-	Wang et al. (2016)
Surface texturing	Cicada wing surface microstructure	Exhibited inhibition *Pseudomonas aeruginosa*	-	Wang et al. (2017)
	Willow-like ZnO nanosheets	Demonstrated high efficacy against gram-positive bacterial	Demonstrated high efficacy against gram-positive bacterial	Ye et al. (2019)
	Zn, oxygen plasma immersion ions, carbon fiber	Discovered to have excellent antibacterial properties against *Staphylococcus aureus* and *Staphylococcus epidermidis*	-	Lu et al. (2016)

### 2.1 Antibiotics loading

The initial stage in the pathophysiology of foreign body infection is thought to involve bacterial adhesion to the surface of biological material (Zilberman and Elsner, 2008; Delaney et al., 2019; Thabit et al., 2019). Following adhesion, certain strains may form BBF if provided with adequate nutrition, which can serve to protect bacteria from immune responses (Davies, 2003; Hall and Mah, 2017). The incorporation of antibiotics into PEEK materials can enhance their antibacterial properties, biological activity, and bone integration to a limited degree. This is primarily achieved through mechanisms of antibiotics such as destruction of cell wall, inhibition of protein synthesis, suppression of peptide synthetase, and other antibacterial effects (Montanaro et al., 2008; Zhang et al., 2022).

#### 2.1.1 Destruction of cell wall

Vancomycin (Van), a glycopeptide antibiotic, can inhibit the synthesis of bacterial cell walls. This inhibits bacterial reproduction and proliferation. Van has emerged as an efficacious antibiotic for the treatment of drug-resistant *cocci* infections, owing to its ability to eliminate a vast majority of gram-positive bacteria and various drug-resistant bacteria (Chen et al., 2021a). Van with slow-release mechanism was built on the surface of PEEK to enhance the efficacy and biosafety of Van-modified PEEK. In contrast, it was reported that Van-loaded gelatin nanoparticles possessed gradual degradation process on PEEK. The degradation process had been found to impede the growth, reproduction, and colonization of *S. aureus*, thereby the potential risk of infection was reduced. Chen et al. grew a Zn oxide nanorod array as an antibiotic carrier on a PEEK substrate (Chen et al., 2019). The Zn oxide nanorod arrays exhibited distinct affinity towards ampicillin (Amp) and Van due to their disparate molecular structure, leading to significantly reduced loading capacities for Amp as compared to Van. Compared to Van, the release rate of Amp was greater. However, bacterial investigations demonstrated that Amp exhibited greater efficacy as a surface modification compared to Van against *S. aureus*.

#### 2.1.2 Inhibition of protein synthesis

**FIGURE 2:**
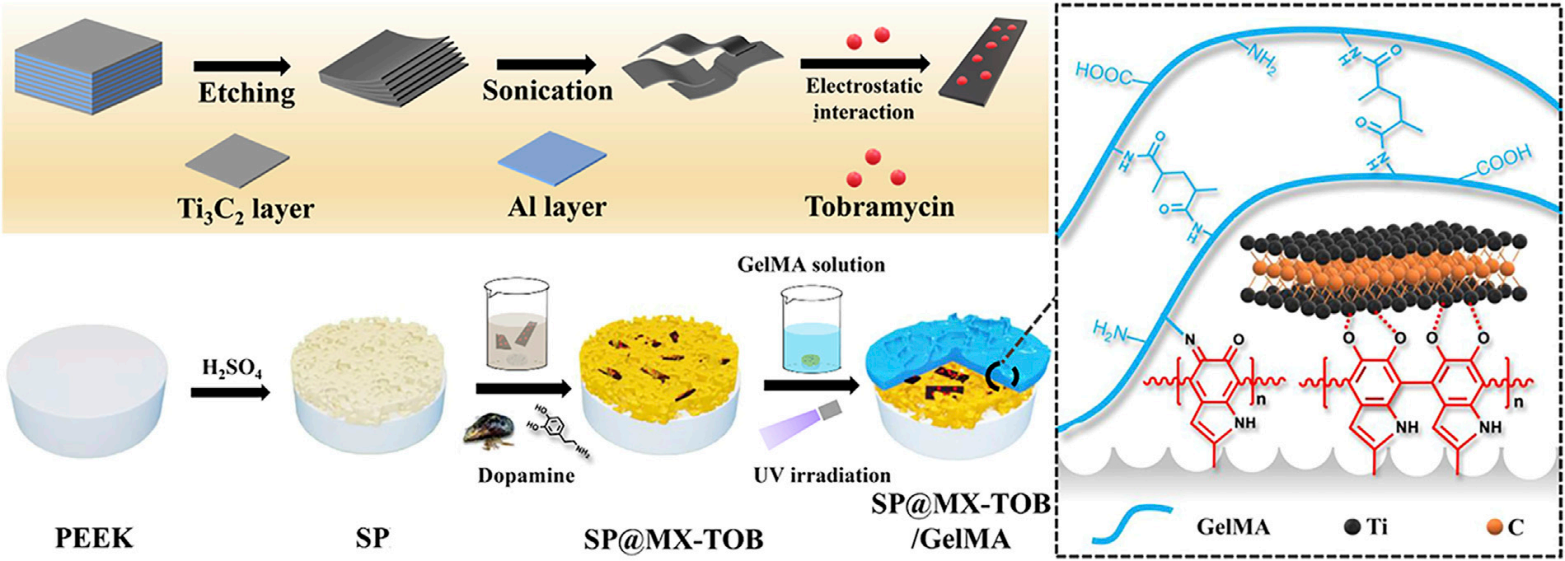
Schematic presentation of the preparation of the multifunctional tissue substrate (SP@MX-TOB/GelMA) (Yin et al., 2020).

Gentamicin sulfate (GS) and tobramycin (TOB) are aminoglycoside antibiotics. TOB inhibits the synthesis of bacterial proteins. Polydopamine (PDA) possesses good adhesion and antioxidation (Liu et al., 2016), which can denature the protein of the cell membrane, destroy the structure of bacterial cell membrane and lead the bacteria to death. TOB was loaded onto MXene nanoplates *via* PDA coating, followed by its combination with gelatin methacrylate (GelMA) and subsequent application onto the biologically inert sulfonated PEEK (SPEEK) (Yin et al., 2020). As shown in Figure 2, Yin et al. (2020) designed a PEEK implant with multiple therapeutic modalities using MXene and GelMA in conjunction with TOB (SP@MX-TOB/GelMA). The photothermal effects of PDA and MXene nanoparticles was observed to facilitate the destruction of both bacteria and osteosarcoma cells. Upon loading TOB, the implants displayed strong antibacterial properties against gram-negative and gram-positive bacteria. The structural composition of GelMA’s arginine-glycine-aspartic acid sequence was found to have a significant impact on stem cell differentiation into osteoblasts, thereby promoting bone regeneration. Additionally, it was demonstrated that MC3T3-E1 cell adhesion and diffusion was significantly enhanced, while also a sterile environment for bone repair procedures in orthopedics was provided. Due to the similar inorganic composition of bone and CaHPO4·2H2O (CaP), CaP-treated PEEK promoted bone regrowth around the implant. To address the issue of low sensitivity to bacteria exhibited by PEEK and CaP, Xue, et al. employed the use of layer-by-layer (LBL) technology to regulate the number of coating layers (Xue et al., 2020). This involved loading GS and CaP onto the material’s surface, resulting in the optimization of both the antibacterial and osseointegration abilities of the modified PEEK. The results demonstrated the antibacterial activity and bone integration capability of PEEK/CaP-GS. PEEK/CaP-GS*6, which was produced through six LBL cycles, exhibited the most potent antibacterial and osseointegration activity. In addition, it was observed that the utilization of PDA and GS SPEEK implants effectively impeded bacterial proliferation and exhibited anti-inflammatory characteristics, thereby rendering them a viable therapeutic option for managing infectious bone defects (Sun et al., 2021).

The simultaneous administration of multiple medications and the exploitation of the combined effects of antibiotics potentially enhanced the antibacterial and biocompatible properties of PEEK. Xu et al. (2019) used a PDA-assisted deposition technique to coat the surface of PEEK with liposomes containing dexamethasone (Dex) and Minocycline (Mino) (Dex/Mino liposomes). The aim was to augment the antibacterial, anti-inflammatory, and osseointegration properties of the material, while also regulating cellular inflammatory response and inhibiting bacterial growth *in vitro*. The utilization of PEEK with co-administration of diverse antimicrobial agents had been shown to enhance the osseointegration and antibacterial efficacy of biomaterials, which exhibits considerable clinical potential.

#### 2.1.3 Suppression of peptide synthetase

β-lactam is a sort of antibiotics by inhibiting the bacterial cell wall non-ribosomal peptide synthetase (Gaudelli et al., 2015). Besides, inhibiting the synthesis of cell wall mucin, which causes bacterial cell wall defects as well as bacterial cell expansion and lysis, will not have a systematic impact on the natural bone tissue. Lactam-based organic antibacterial membrane materials were developed by dissolving SPEEK in dimethyl sulfoxide containing lactam drugs. It also efficiently inhibited the reproduction and growth of *S. aureus* while maintaining outstanding mechanical properties (Montero et al., 2016).

However, the extensive use of antibiotics has contributed to the emergence of superbugs as well as the development of bacterial antibiotic resistance (Raphael and Riley, 2017; Singh et al., 2017; Yilmaz and Ozcengiz, 2017). The development of drug-resistant bacteria surpasses the development of new medicines, and drugs are expensive (Li et al., 2018b). Additionally, antibiotics may have negative effects on the host (Spellberg et al., 2008; Yilmaz and Ozcengiz, 2017). Therefore, it is imperative to develop antimicrobial drugs with high efficacy and low toxicity while preventing the emergence of novel resistant bacteria and the side effects of conventional antibiotics.

### 2.2 Animal and plant extracts

The abuse of antibiotics leads bacteria to develop multiple antibiotic resistance. Antimicrobial peptides (AMPs) and plant polyphenols have received a great deal of attention and research in recent years because of their clinical application potential for antimicrobial resistance and biocompatibility.

#### 2.2.1 AMPs

Almost all organisms contain natural AMPs, which has an extensive range of structures and functions. AMPs are not toxic to mammalian cells, and have low drug resistance to microbes. Most AMPs at neutral pH are cations. Contrarily, gram-positive bacteria have negatively charged phosphate groups on the surface of their cell walls, as well as gram-negative bacteria have an abundance of negatively charged lipopolysaccharide in their cell membranes (Yan et al., 2021b). Positively charged AMPs can bind to bacteria with negative charge, perform membrane-destructive activity, and directly kill microbial pathogens *via* electrostatic contact. By regulating the immune response of the host, AMPs assisted in the indirect elimination of the pathogen from the host (Ouyang et al., 2016). Some AMPs also exhibited osteogenic activity with their extensive antibacterial properties (Li et al., 2021; Yuan et al., 2021).

Bone marrow mesenchymal stem cells (BMSCs) could proliferate osteogenically when the BMP/Smad signaling pathway was stimulated by KR-12 (Li et al., 2018a). The positively charged amine group in the amino acid sequence of antimicrobial peptide KR-12 attracted negatively charged bacteria to the surface, where the cell walls had been changed and demolished. PEEK coated with KR-12 improved antibacterial activity against *S. aureus in vitro* and *in vivo* (Meng et al., 2020). To increase antibacterial properties and bone integration, Yuan, et al. coated the surface of 3D porous SPEEK with mouse beta-defensin-14 (MBD-14) (Yuan et al., 2019). It was structurally and functionally analogous to human beta-defensin-3 (HBD-3) (Hinrichsen et al., 2008; Rohrl et al., 2008). MBD-14 was effectively loaded onto the porous SPEEK surface following a freeze-drying treatment and exhibited long-term antibacterial activity against gram-positive and gram-negative microorganisms. MBD-14-loaded SPEEK could efficiently stimulate osseointegration and protein expression (Yuan et al., 2019).

Hu et al. (2021) and colleagues used a wet chemical technique to coat GL13K, which was a kind of AMPs, directly on the PEEK surface for the first time. To induce binding, GL13K was exposed to 1-ethyl-3-(3-dimethylaminopropyl) carbodiimide (EDC) impregnated with the PEEK matrix. The procedure could effectively improve hydrophilicity, increase antibacterial activity (with a 28-mm inhibitory zone), flatten the surface, decrease surface roughness.

AMP and AMP-based molecules are promising antibiotic alternatives. Their distinct antimicrobial defenses are an effective tool against the threat posed by drug-resistant bacteria. Unfortunately, there are still numerous issues with AMP development and implementation that must be addressed. The study of AMP toxicity and structure is currently in progress, and release kinetics of AMPs *in vivo* are insufficient. Research in this field is still primarily focused on better AMP stability, higher selectivity, and reduced toxicity. Developing an effective dosing schedule, combining AMPs with other compounds to increase their antibacterial efficacy, and other measures will be necessary for the clinical application of AMPs (Lazzaro et al., 2020; Yan et al., 2021b).

#### 2.2.2 Plant polyphenols

Plants contain natural biological compounds known as plant polyphenols. Approximately 500 of the over 8,000 plant polyphenols have biological effects (Nicolin et al., 2019), which contain antioxidant, anti-inflammatory, and bone-growth-promoting characteristics. Hinokitiol (HK) is a naturally occurring plant polyphenol with anticancer, anti-inflammatory, anti-bacterial, and antioxidant activity (Hajjaji et al., 2007). Zhang et al. (2018) found that HK-loaded nano-bioglass/PEEK composites exhibited potent antibacterial activity against *S. aureus*, had no appreciable effect on MC3T3-E1 cell adhesion and cell replication, and did not significantly promote bone formation *in vivo*. Lawsone, a 2-hydroxy-1,4-naphthoquinone, presents in the nail mosaic extract, with efficacious function against gram-positive bacteria (Ur Rehman et al., 2018). Due to its rough and honeycomb structure, PEEK treated with bioactive glass provided delayed and prolonged release of lawsone for up to 6 months. Furthermore, researchers conducted extensive research to identify a wider range of antimicrobial plant polyphenols. Resveratrol (RV) has been identified in several plants, including red wine, grapes, peanuts, and fruits (Karimi-Soflou et al., 2022). By using femtosecond laser technology, RV was encapsulated in the nano-porous structure of a PEEK mixed with nano-porous magnesium calcium silicate composite. It was subsequently showed remarkable antibacterial effect of the above composite against *E. coli* and *S. aureus* by activating the ERK/MAPK and Wnt/β-catenin signal pathways, which was associated with adhesion, replication, induction of stem cell differentiation into osteoblasts, and osteogenic gene expression of BMSCs. For further intensive research, the pattern of nano-porous tantalum pentoxide particles with pore diameters of approximately 10 nm and PEEK (PN) was carefully prepared by cold pressing and sintering (Mei et al., 2021). The PN was then soaked in concentrated sulfuric acid to shape a micropore structure on the sulfonated PN’s surface (SPN). Finally, the porous surface of SPN was coated with genistein (SPNG), which was exhibited excellent antibacterial activity and inhibited the growth of *E. coli* and *S. aureus in vitro* because of the synergistic interaction between the -SO_3_H group and the continuous release of genistein. Plant polyphenol chlorogenic acid (CGA) was predominantly extracted from *Fols Lonicerae*. In order to solve the problem of large area defect and related infection in clinical bone grafting, CGA was loaded on the surface of SPEEK using a hydrogel system (He et al., 2019). Following the initial outbreak, CGA was continually released in the hydrogel system for 8 days with suppressing *E. coli* and *S. aureus* and minimizing the probability of early implant infection. Curcumin (CR) is a polyphenol compound produced by turmeric extract that inhibits the replication of bacteria and fungi as well as inflammation and oxidation (Zou et al., 2020). CR and GS were loaded on the PEEK surface to enhance the osseointegration around implants and reduce implant infection by continuous release of GS and CR with the cumulative emissions of GS and CR were finally all greater than 60%. Together, Modified PEEK prevents the reproduction of *S. aureus* and *E. coli*.

### 2.3 Loaded metal ions

The antibacterial effects of metal ions are generally accomplished by rupturing bacterial cell walls, developing cell ulcers, and killing the bacterium. Metal ions penetrate the bacteria and attack the nucleus, causing denaturation of bacteria and eventually cell death (Chudobova et al., 2015; Yan et al., 2018b; Cyphert et al., 2021). Therefore, the growth and proliferation of bacteria were markedly inhibited. Additionally, it is possible to significantly improve the antibacterial properties and biocompatibility of modified PEEK materials through the dual loading of antibacterial metal ions and pharmaceuticals or synergistical employment of various metal ions.

#### 2.3.1 Ag

It is reported that Ag has high catalytic ability due to its chemical structure (Farkas et al., 2010). The reduction potential is observably increased in high oxidation state of Ag, which is in favor of sterilization by production of atomic oxygen.

Seuss et al. (2016) used electrophoretic deposition for fabricating bioactive glass and Ag nanoparticles (NPs) composite coatings on the PEEK surface. It was suggested that involvement of PEEK substrate facilitated the uniform distribution of bioactive glass particles, while the electrophoretic deposition process unaffected by Ag NPs remained for ensuring the antibacterial properties of the coating against *E. coli*. Deng et al. (2017) prepared Ag NPs loaded onto the 3D-printed PEEK surface using PDA-assisted deposition. According to the findings, PEEK scaffolds coated with Ag NPs dramatically reduced the proliferation of gram-positive and gram-negative bacteria. Contact killing and release killing of Ag NPs were primarily responsible for the antibacterial impact, which also eliminated any existing BBF. Furthermore, the presence of Ag NPs did not affect the division and differentiation of osteogenic cells, which exerted no influence on bone metabolism. Furthermore, magnetron sputtering was successfully employed by Liu et al. (2017) to deposit dense and uniform coating of modified Ag NPs on the surface of PEEK. Under the acceleration of electric and magnetic fields, the excited results on PEEK surface etching of Ag were demonstrated. Ag was also polymerized on the surface of the material, producing nano-scale surface morphology. The increase of Ag modified PEEK roughness improved the adhesion of PEEK implants to bone *in vivo*. Ag NPs adhered to the surface of bacteria directly penetrated into cells, and eventually caused cell death without damaging the cell membrane. Consequently, the Ag NPs modified PEEK implants evidently increased bacterial adhesion and antibacterial activity while exhibiting negligible cytotoxicity.

With demonstration of further in-depth research, Ag combined with antibiotics exhibited more remarkable antibacterial activity and widely expansion of antibacterial spectrum (Morones-Ramirez et al., 2013). As a result, developing a system using biomedical devices, which are suitable for orthopedic implantation, to achieve on-demand simultaneous release of Ag and antibiotics is the better alternative. Therefore, Ag NPs with silk fibroin (SF)/GS coating on the porous SPEEK surface was prepared (Yan et al., 2018a). In contrast to SPEEK blended with either GS or Ag NPs alone, it was explored that SPEEK loaded with Ag NPs and GS exhibited superior antibacterial properties. The observed results demonstrated that the synergistic interaction of Ag NPs and GS enhanced the antibacterial potential. In addition, Yu et al. (2021) developed bone forming peptide (BFP) loaded PEEK with a layer of carboxymethyl chitosan (CMC) film on the surface by using spin coating. The surface of PEEK was then loaded with Ag NPs using dopamine self-polymerization. The results demonstrated that PEEK coated with Ag NPs exhibited an outstanding inhibitory effect on bacteria. The surface release of Ag ions can be controlled by using CMC films. PEEK loaded with BFP was more physiologically active than pure PEEK and promoted osteogenic differentiation and cell proliferation.

#### 2.3.2 Cu

Ag is widely involved in clinical antibacterial therapy. However, certain bacteria that are resistant to silver have been observed (Gupta et al., 1999; Mijnendonckx et al., 2013). Therefore, it is imperative to discover a new agent that can effectively kill bacteria without developing drug resistance. Cu exerts bactericidal activity through the disruption of bacterial proteins as well as the production of reactive oxygen species (ROS). Liu et al. (2019) developed a method to immobilize copper NPs with antibacterial properties onto the surface of SPEEK biomaterials. This was achieved by creating a porous microstructure to trap *S. aureus* on the surface of PEEK biomaterials by sulfonation. The results indicated that the porous surface of Cu NPs adsorbed on SPEEK could effectively capture *S. aureus*. Additionally, the release of Cu from SPEEK-immobilized Cu NPs could potentially induce an increased polarization of macrophages, which resulted in promotion of *S. aureus* phagocytosis and antibacterial efficacy. Yan et al. (2021a) utilized PDA-assisted deposition to load Cu citrate nanoclusters onto porous SPEEK surfaces. The permeability into bacterial membrane of citrate was dominate in bactericidal activity. Research findings suggested that the application of Cu citrate complex as nano-antibiotic exhibited superior efficacy in the degradation of bacterial membrane as compared with either Cu or citrate alone. Collectively, their synergistic effect played a crucial role in the complete degradation of proteins and induction of bacterial mortality. In addition, it caused a local increase in the concentration of Cu in the bacteria. Increased local Cu concentration increased intracellular ROS production by catalyzing the Fenton reaction and the formation of hydroxyl radicals, which was beneficial in DNA damage and suppression of particular enzyme activity. Interestingly, Yan et al. (2020) investigated the fabrication of CuO microspheres with Ag NPs on the surface of porous PEEK with silk fibroin. PDA-assisted deposition of Ag NPs on CuO microspheres built development of materials that exhibited remarkable antibacterial properties. It was indicated that the antibacterial efficacy of CuO/Ag NPs was influenced by pH, as evidenced by significant increase in the release of Cu and Ag at lower pH values, resulting in a greater bactericidal effect compared to SPEEK with either Ag or Cu alone. Apparently, it was an effective method to synergistically load antibacterial metal ions and bioactive ceramics on the surface of PEEK materials to realize the biofunctionalization of antibacterial properties and bioactivity of PEEK implant materials. However, it is worthless that Ag and Cu may exhibit cytotoxic effects, which tend to increase with the concentration of metal ions (Vimbela et al., 2017). How to optimize cytotoxicity and antibacterial properties will be a focus of future research.

#### 2.3.3 Mg

Nanosized Mg and its alloys have been reported to exhibit anti-inflammatory and antioxidant activity (John Sushma et al., 2016). Mg can induce the formation of ROS, which cause lipid peroxidation in the microbial cell envelope, resulting in the outflow of cytoplasmic content from the cell (Blecher et al., 2011). Yu et al. (2018) used physical vapor deposition to deposit Mg loading on the surface of PEEK. The findings indicated that the application of Mg coating participated in significant bactericidal effect against *S. aureus*, with antibacterial efficacy of 99%. It followed that the application of Mg coating PEEK exhibited remarkable antibacterial characteristics. Simultaneously, the application of Mg coating has been observed to considerably enhance the biological activity of PEEK. Ti/Mg/Ag gradient composite coatings were prepared on the surface of PEEK by magnetron sputtering (Gümüş et al., 2017). It was suggested that the incorporation of Ti bottom layer significantly strengthened the elastic modulus and bone conductivity of PEEK. Additionally, the middle layer composed of Mg improved biological activity, and the surface layer of the antibacterial agent composed of Ag provided the antibacterial ability of the preparation. Moreover, Niu et al. (2020) investigated the antibacterial and biological properties of PEEK/nano-Mg silicate composites. The modified PEEK composites exhibited notable antibacterial properties against *E. coli* and *S. aureus*, in addition to significant enhancement of the adhesion, diffusion, proliferation, and differentiation of BMSCs.

According to the studies above, antimicrobial metal ions modification promoted antibacterial activity of PEEK and its derivant, although this is far from having the desired antibacterial potential. Based on this, the co-loading of a variety of antibacterial ions and the synergistic effect between antibacterial metal ions provided further improvement of the antibacterial and biocompatibility of PEEK.

### 2.4 Photothermal catalyst

Using photothermal catalyst with catalytic oxidation and thermal effect to enhance the antibacterial performance of PEEK implant materials has recently become a major area of research.

**FIGURE 3:**
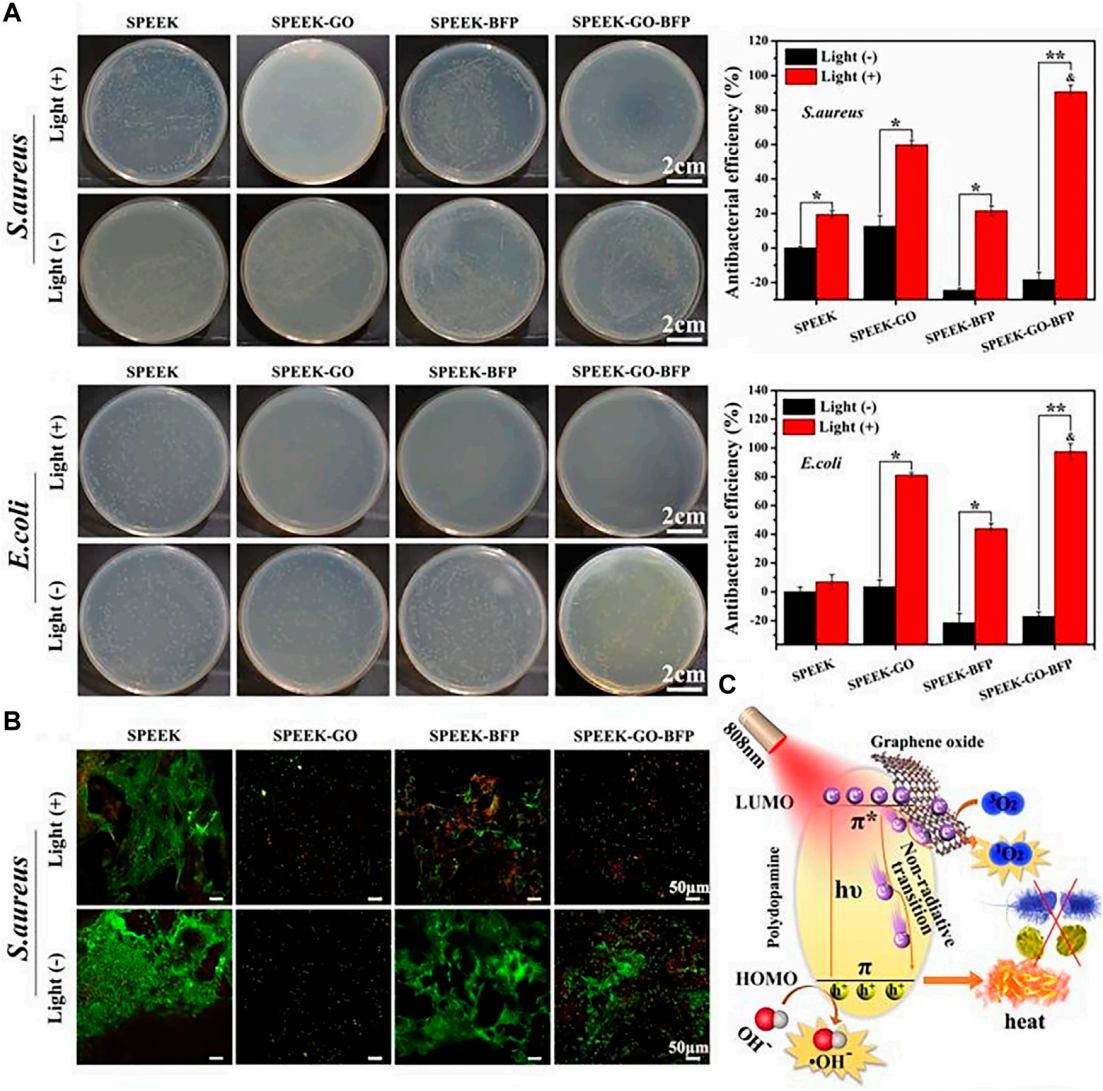
Antibacterial activities of the 2D nano-coatings with or without light: **(A)** The spread plate images with antibacterial efficiency, and **(B)** Live/Dead staining of Gram-positive/negative bacteria. **(C)** Schematic diagram of antibacterial phototherapy through combined photodynamic therapy and photothermal therapy (Wang et al., 2020).

Graphene oxide (GO), a type of photothermal catalyst provided with unique single-layer carbon atomic nanosheet structure and excellent intrinsic antibacterial effect (Zou et al., 2017), has been shown to effectively mitigate cytotoxicity, enhance cytocompatibility, and facilitate the process of stem cell osteogenesis both *in vitro* and *in vivo* (Qin et al., 2020). According to studies, GO wrinkles reduced bacterial biological activity and, as a result, had effective antibacterial activity (Ouyang et al., 2018a). Ouyang, et al. used a simple dipping method to prepare GO-modified SPEEK, which demonstrated improved antibacterial activity and cytocompatibility. It was predominantly attributed to the presence of GO, which enhanced the antibacterial properties and biocompatibility of materials against *E. coli*, meanwhile sulfonated porous micro/nanostructures provided more sites for GO loading and promotion of cell proliferation and differentiation. Simultaneously, porous surfaces of SPEEK were fabricated by Wang et al. (2020) through the assembly of GO nanosheets, PDA nanofilms, and oligopeptides. The results indicated that the novel nano-coating with multiple functions had the potential to enhance cytocompatibility and bone formation *in vivo*. The application of mixed coating of GO/PDA on SPEEK samples also resulted in higher production of ROS after irradiation, in comparison to the application of single coating of either GO or PDA. The synergistic effect of photothermal/photodynamic therapy was attributed to the mechanism of antibacterial activity of GO/PDA two-dimensional nano-coating, as shown in Figures 3A,B. PDA possessed inherent near-infrared absorption capabilities, rendering it promising natural pigment for augmenting light-capturing potential of GO nanoparticles. As shown in Figure 3C, *π*-π stacking GO/PDA hybrid complex anchored on the surface of SPEEK has been found to generate potent antibacterial phototherapy effect through synergistic action of photothermal and photodynamic therapy. It was observed that GO impeded the proliferation of bacteria. Bacterial membrane bore mechanical stress on the sharp edge of GO, leading to cellular disintegration. Simultaneously, GO caused oxidative stress from ROS, leading to detrimental effects on the DNA and structural integrity of bacteria. Hence, the application of GO coating is considered a promising approach to enhance the cytotoxic and antibacterial properties for modified PEEK implant. Qin et al. (2021) produced a composite coating of GO, carbon fiber (CF), and PEEK (GO/CF/PEEK) using electrostatic powder spraying, which was subsequently applied onto the surface of Ti-6Al-4V alloy. Due to the potential of GO to generate ROS and antibacterial properties by destroying bacterial DNA and its expressed protein, the study found that the GO/CF/PEEK coating exhibited a remarkable inhibitory effect on *S. aureus*.

Furthermore, the antibacterial activity and biocompatibility of PEEK were significantly improved by synergistic effect of photothermal catalysts and antimicrobial agents. Ouyang, et al. (Ouyang et al., 2018b) modified SPEEK by coating its surface with PDA, and then with GO/Dex-loaded liposomes to produce GO/Dex-modified SPEEK. It was demonstrated that GO and hydrophilic groups at the liposome’s edge, such as -OH and COO-, absorbed proteins *via* electrostatic interaction, controlled cell adhesion, and stimulated specific gene expression. Consequently, the synergistic effect of GO/Dex-loaded liposomes increased cellular adhesion and migration, as well as improved cellular biocompatibility.

It was reported that GO in the range of 100–200 nm was satisfied with the majority of the requirements of successful drug carrier. However, GO was rarely characterized by uniform particle sizes, which might have significant impact on its biocompatibility *in vitro* and *in vivo*. Meanwhile, bio-persistence of GO in the human body might have an impact on pathology and immunology (Gallo et al., 2014). As a result, the next challenges entail establishing the optimal dosage that achieves equilibrium between the therapeutic effects and nanotoxicity of GO-based formulations. Additionally, standardizing the dosage employed in GO research, and elaborating on the principle of toxicity and its remedies are necessary to yield comparable outcomes and elucidate the potential cytotoxicity effects of GO-based formulations.

### 2.5 Doped bioactive coating

Bioactive coating, the application of bioactive materials in the field of PEEK modification, provide interaction between PEEK and surrounding micro-environment (Barrere et al., 2003). These bioactive materials not only possess good biocompatibility and bone conductivity, but also degrade and release ions *in vivo*, forming a weak alkaline environment to improve cell activity and accelerate bone repair (Pan et al., 2013). Certainly, bioactive coating is helpful for antibacterial behavior *via* disintegrating the bacterial cell membrane.

Chen et al. (2017) applied argon plasma immersion ion implantation and hydrofluoric acid (Ar-PIII) treatment to introduce fluorine onto the surface of PEEK. It was observed that the fluorinated PEEK strengthened the activity of alkaline phosphatase (ALP), as well as promoted cell adhesion, replication, and proliferation, which was beneficial to inhibitory efficacy on *Porphyromonas gingivalis*, recognized as one of the primary pathogens of periodontal disease. Wang et al. (2014) created PEEK and nano-fluorinated hydroxyapatite (n-FHA) biological composites. It was indicated that PEEK/n-FHA biological composites exhibited increased cell adhesion and proliferation *in vitro*. Moreover, cells cultivated on PEEK/n-FHA biological composites showed increased ALP activity and cell mineralization, which substantially enhanced osseointegration capacity. Importantly, it was observed that the PEEK/n-FHA biological composites showed promise ability for reducing bacterial expansion and BBF development.

Wang et al. (2016) used a simple chemical precipitation method to deposit red Se NPs onto the surface of PEEK. The conversion of red Se NPs to gray Se NPs under heating was observed to result in a modified PEEK surface with notable antibacterial properties. After 3 days, it was demonstrated that PEEK coated with red or gray Se exhibited significant inhibition of *P. aeruginosa* growth in comparison to uncoated PEEK. The surface of PEEK coated with either red or gray Se resulted in reduction of its hydrophilicity and played a crucial role in the prevention of bacterial proliferation.

### 2.6 Surface texturing

To reduce the issues associated with excessive antibiotic usage, researchers have focused on altering the shape, size, and distribution of material surfaces to influence the biological behavior of bacteria. This approach has gained attention as an attractive option for enhancing the antibacterial properties of PEEK implant materials without the need for additional antimicrobial agents. The surface structure of the column on the cicada’s wing provided sterilization of the gram-negative bacteria, a purely physical mechanism, not a chemical process (Ivanova et al., 2012).

Wang et al. (2017) used 3D printing technology to create PEEK materials with cicada wing texture surfaces. The research findings indicated that PEEK with cicada wing surface microstructure exhibited superior antibacterial properties as compared to conventional PEEK materials in the orthopedics. The amount of *Staphylococcus epidermidis* discovered on the surface of PEEK also decreased by more than 37%. Following single day of cultivation, the adhesion and growth of *P. aeruginosa* experienced a reduction of 28%. Subsequently, after 5 days, a reduction of approximately 50% was observed. It was demonstrated that the potential of nanostructured PEEK by physical method showed impressive antibacterial properties, such as by texturing the surface of PEEK, rather than through the use of antimicrobial agents or antimicrobials.

According to Ye et al. (2019), the surface microstructure with single surface texture of cicada wing might be ineffective against gram-positive pathogens such as *S. aureus*. An additional bionic structure comprising willow-like ZnO nanosheets was incorporated into the existing structure to compensate for the lack of single biomimetic component. The nanosheets exhibited rapid release profile upon initial implantation and high efficacy against gram-positive bacterial infections. The advancement of dual bionic materials could potentially enhance the efficacy of clinical material designs in the future. Lu et al. (2016) implanted Zn and oxygen plasma immersion ions onto the surface of CF to modify the surface of PEEK by incorporating Zn and introducing unique micro/nanostructures. The multi-stage Zn/O structure of CF-enhanced PEEK was discovered to have excellent antibacterial properties against BBF-positive bacteria, including *S. aureus* and *S. epidermidis*. However, the antibacterial efficacy against BBF-forming gram-negative bacteria was limited. A plausible explanation was that the Zn/O-modified PEEK surface formed microstructure similar to that of bacteria. Consequently, the microstructure created micro-pits that separated and confined bacteria exhibiting positive BBF, finally minimized their contact with neighboring bacteria. This process effectively enhanced antibacterial efficacy by impeding the development of BBF.

Surface texturing represents a novel method for the production of antibacterial surfaces and an alternative to chemical-based methods. However, narrow antibacterial spectrum and slow inhibition of bacterial proliferation limit further application.

## 3 Conclusion and future look

Due to remarkable chemical stability, low biological repellency, and similar Young’s modulus comparable to human bone tissue, the application of PEEK represents the popularity of novel orthopedic material and carrier pattern. The utilization of antimicrobial modified PEEK material has been observed to effectively impede bacterial colonization and reproduction. The present study systemically summarized the methods and mechanisms employed for surface modification of PEEK and its composites for improvement of anti-infection properties in the field of bone infection. In fact, many modification ways including antibiotics, animal and plant extracts, metal ions, photothermal catalyst, bioactive coating, surface texturing and so on provided superior antibacterial behavior and osseointegration ability in the territory of designation of PEEK anti-infection orthopedic implants. The addition of antibiotics was primarily intended to achieve antibacterial effect through various mechanisms. However, the overuse of antibiotics was responsible for the development of multiple drug resistance in bacteria, while the discovery of AMPs effectively prevented the abuse of antibiotics. Because of favorable biocompatibility, low cytotoxicity, and lack of drug resistance, scientists have become increasingly concerned about AMPs in recent years. Similarly, plant polyphenols possessed antioxidation, anti-inflammatory, and bone-immunity properties, which enabled develop the PEEK-modified materials with multiple functions simultaneously. The researchers were attempting to determine the optimal dosage of biomolecules to prevent excessive adverse effects. By the judicious design of biomolecule sustained release system, it was also observed that biomolecules were increasingly employed in the surface coating. At the same time, the antibacterial properties of metal ions were gradually much attention for their ability to disrupt the bacterial cell wall, generate cell fester, and cause bacterial destruction. Furthermore, the synergistic efficacy of metal ions and drugs extremely enhanced the antibacterial properties. Furthermore, through the involvement of the photothermal catalyst’s catalytic oxidation effect and surface texture control, the material surface possessed antibacterial properties without the addition of antimicrobial agents, which has become a hot research direction for the antibacterial properties of PEEK-modified materials.

At present, the main treatment of bone infection is staged operation. To minimize the physical, psychological, and economic burden of patients, it is believed that the staged operation should evolve into a one-stage operation. This requires internal fixator to promote bone integration while being antibacterial, so the anti-infective PEEK internal fixator emerging as an effective strategy in the current research. Further, the use of two or more modified PEEK with bio-inert has been investigated to explore more possibilities. However, considering that PEEK as orthopedic implant material will coexist with natural tissues for long period, the scope and depth of its research must be expanded. It is suggested the following characteristics: 1) Strategies including strong acid sulfonation, and 3D printing make sure the enhancement of the surface roughness of PEEK, augmentation of the hydrophilicity and promotion of the adhesion of osteocyte and bacteria. 2) Establishment of optimal dose and the appropriate controlled-released system to achieve the balance between therapeutic efficacy and cytotoxicity. 3) According to the characteristics of bone infection, such as dead cavity, local infection, bone defection, and so on, multifunctional formulation with antibacterial, osteointegration, and osteogenesis is also an important future direction of modified PEEK in bone infection.
