# Supplementary material for: Research progress and future prospects of antimicrobial modified polyetheretherketone (PEEK) for the treatment of bone infections
Source: Front Bioeng Biotechnol. 2023 Aug 3;11:1244184. doi: 10.3389/fbioe.2023.1244184 (PMC10436002; doi:10.3389/fbioe.2023.1244184)
Supplement: Supplementary file 1 [file Table1.DOCX]

**Supplementary notes of figures**


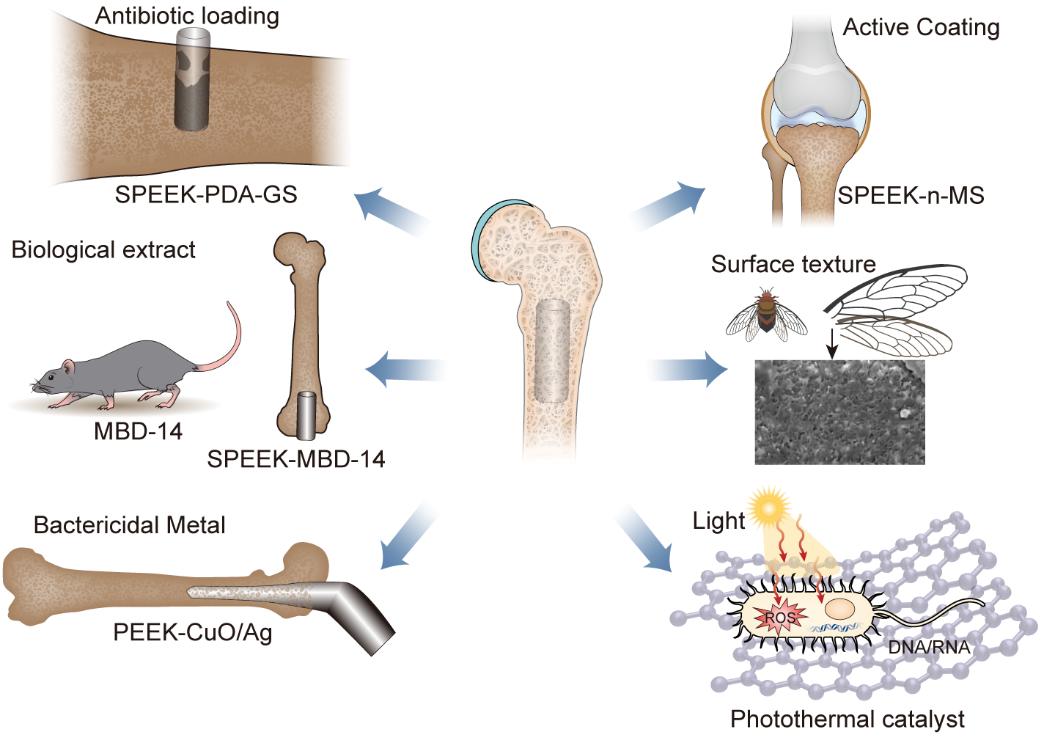


**Fig. 1.** The main classification of antibacterial surface modification of PEEK, including load antibiotics, biological extract, bactericidal mental, active coating, surface texture, and photothermal catalyst. **Abbreviation:** SPEEK, sulfonated polyether ether ketone; GS, Gentamicin sulfate; MBD-14, mouse-defensin-14; n-MS, nano-magnesium silicate


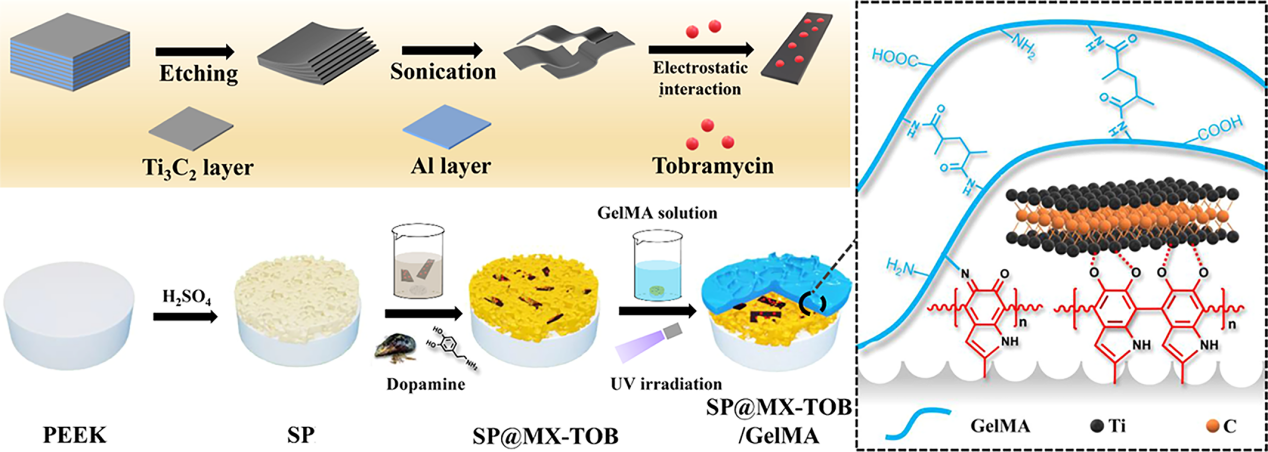


**Fig.2.** Schematic presentation of the preparation of the multifunctional tissue substrate (SP@MX-TOB/GelMA) (Yin, et al. 2020)

**
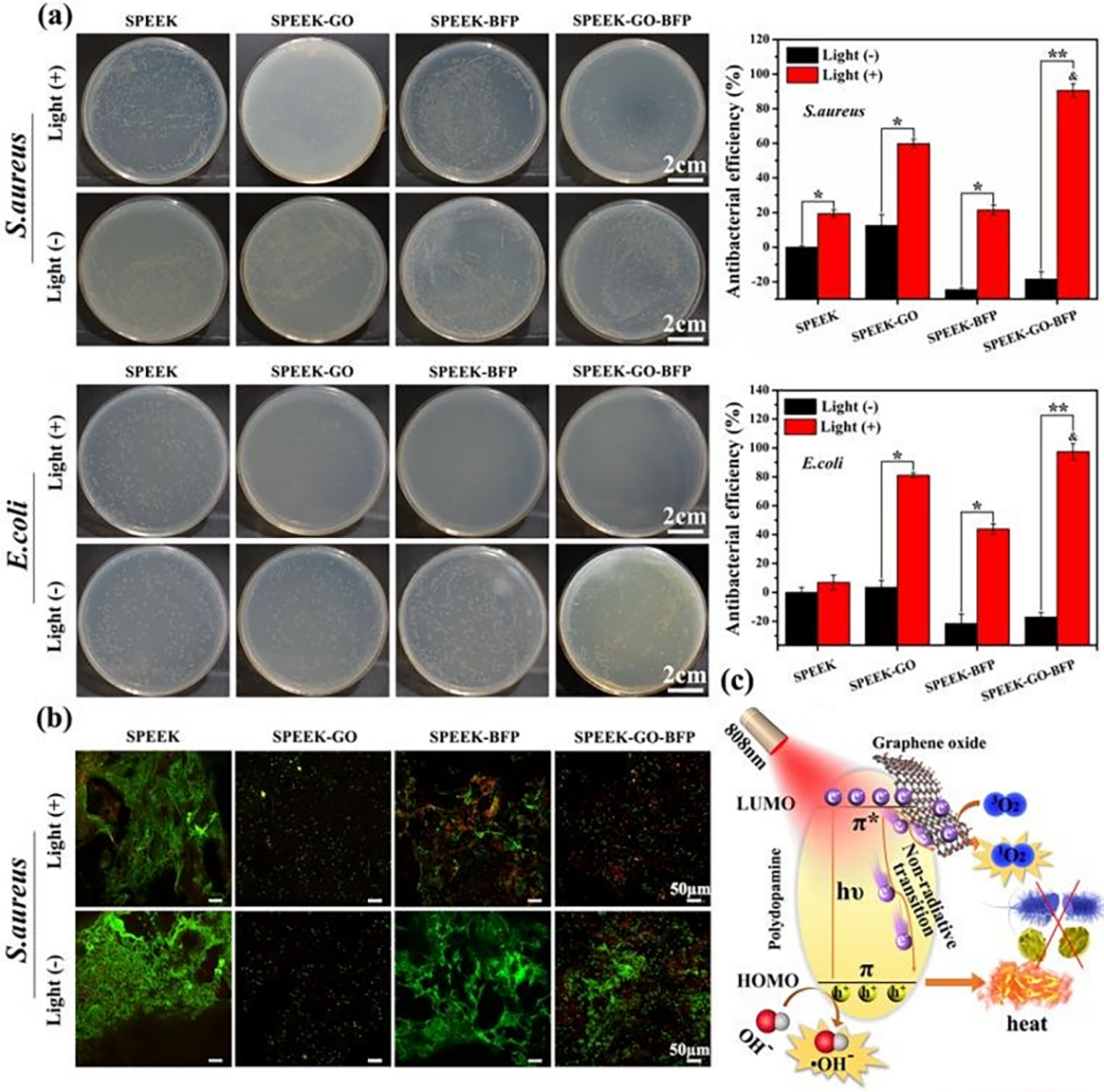
**

**Fig.3.** Antibacterial activities of the 2D nano-coatings with or without light: (a)The spread plate images with antibacterial efficiency, and (b) Live/Dead staining of Gram-positive/negative bacteria. (c) Schematic diagram of antibacterial phototherapy through combined photodynamic therapy and photothermal therapy (Wang, et al. 2020).
