# Supplementary material for: Research progress and future prospects of antimicrobial modified polyetheretherketone (PEEK) for the treatment of bone infections
Source: Front Bioeng Biotechnol. 2023 Aug 3;11:1244184. doi: 10.3389/fbioe.2023.1244184 (PMC10436002; doi:10.3389/fbioe.2023.1244184)
Supplement: Supplementary file 2 [file Table2.DOCX]

**Table 1.**

Antibacterial modification strategies on PEEK and its composites for antibacterial properties.

| Modification strategy | Modification Coatings | Results related to antibacterial property *in vitro* study | Results related to antibacterial property *in vivo* study | Reference |
| --- | --- | --- | --- | --- |
| Loaded antibiotics | Van, ZnO, Amp | Had good inhibitory effect on the growth of *Staphylococcus aureus*. | - | (Chen, et al. 2019) |
|  | TOB, PDA, GelMA | Defeated osteosarcoma cells and bacteria | Excellent osteogenic activity | (Yin, et al. 2020) |
|  | GS, CaP | Excellent and sustained antibacterial property, biocompatibility and cell osteogenic differentiation | Exhibited in *vivo* antibacterial activity and osseointegration ability in the treatment of bone defect with infection | (Xue, et al. 2020) |
|  | GS, PDA | Impeded bacterial proliferation and exhibited anti-inflammatory characteristics | Excellent osteogenic activity and antimicrobial properties | (Sun, et al. 2021) |
|  | Dex/Mino liposomes | Enhance the osseointegration and antibacterial efficacy of biomaterials | Enhanced osteointegration, and antibacterial  activity in beagle femoral implant models | (Xu, et al. 2019) |
|  | β-lactam antibiotics | Inhibited the reproduction and growth of *Staphylococcus aureus* | - | (Montero, et al. 2016) |
| AMPs | KR-12, PDA | improved antibacterial activity against *Staphylococcus aureus* | Increased  osteointegration in rats, improved antibacterial activity against *Staphylococcus aureus* | (Meng, et al. 2020) |
|  | MBD-14 | Exhibited long-term antibacterial activity against gram-positive and gram-negative microorganisms | Stimulated osseointegration and protein expression | (Yuan, et al. 2019) |
|  | GL13K, EDC | Provided bactericidal activity and biofilm resistance | - | (Hu, et al. 2021) |
| Loaded plant polyphenols | HK, nano-bioglass | Exhibited potent antibacterial activity against *Staphylococcus aureus* | Did not significantly promote bone formation | (Zhang, et al. 2018) |
|  | Lawsone, bioactive glass | Exhibited potent antibacterial activity against *Staphylococcus aureus* | - | (Ur Rehman, et al. 2018) |
|  | RV, nano-porous magnesium calcium silicate | Antimicrobial and  osteogenesis activity | - | (Karimi-Soflou, et al. 2022) |
|  | Genistein, nano-porous tantalum pentoxide, sulfuric acid | Inhibit the growth of *Escherichia coli* and *Staphylococcus aureus* | Increased bone integration, stimulated the response of BMSC | (Mei, et al. 2021) |
|  | CGA, hydrogel | Suppressed *Escherichia coli* and *Staphylococcus aureus* | - | (He, et al. 2019) |
|  | CR, GS | Prevents the reproduction of *Staphylococcus aureus* and *Escherichia coli*. | Accelerated osteogenesis and osseointegration | (Zou, et al. 2020) |
| Loaded metal ions | Ag NPs, PDA | Reduced the reproduction of gram-positive and gram-negative bacteria | - | (Deng, et al. 2017) |
|  | Ag NPs | Increased antibacterial activity | Improves adhesion of PEEK implants to bone | (Liu, et al. 2017) |
|  | Ag NPs, GS, silk fibroin | Exhibited superior antibacterial properties | - | (Yan, et al. 2018) |
|  | Ag NPs, CMC, BFP | Exhibited an outstanding inhibitory effect on bacteria, promoted osteogenic differentiation and cell proliferation | - | (Yu, et al. 2021) |
|  | Cu NPs | Effectively captured MRSA | Effectively captured MRSA | (Liu, et al. 2019) |
|  | Cu nanoclusters, PDA, citrate | Enhanced the bacteria-killing ability | Encouraged bone regeneration of implants | (Yan, et al. 2021) |
|  | CuO, Ag NPs, silk fibroin, PDA | Presented synergistic antibacterial ability, potentiated osteodifferentiation | Increased bone integration, stimulated the response of BMSC | (Yan, et al. 2020) |
|  | Mg | Demonstrated a significant bactericidal effect against *Staphylococcus aureus,* enhanced the biological activity | - | (Yu, et al. 2018) |
|  | Ti/Mg/Ag gradient composite coatings | Improve the antibacterial ability, biological activity and bone conductivity | - | (Gümüş, et al. 2017) |
|  | Nano-magnesium silicate | Exhibited notable antibacterial properties against *Escherichia coli* and *Staphylococcus aureus*, enhanced the differentiation of BMSCs | - | (Niu, et al. 2020) |
| Loaded photothermal catalyst | GO | Enhanced the antibacterial properties and biocompatibility of materials against *Escherichia coli,* promoted cell proliferation and differentiation | - | (Ouyang, et al. 2018a) |
|  | GO nanosheets, PDA, oligopeptides | Impeded the proliferation of bacteria, lead to cellular disintegration | Enhanced cytocompatibility and promoted bone formation | (Wang, et al. 2020) |
|  | GO, carbon fiber, Ti-6Al-4V | Exhibited a remarkable inhibitory effect on *Staphylococcus aureus*, enhanced cytocompatibility | - | (Qin, et al. 2021) |
|  | GO, PDA, Dex- liposomes | Enhanced cellular adhesion and migration, as well as improve cellular biocompatibility | - | (Ouyang, et al. 2018b) |
| Doped bioactive coating | Hydrofluoric acid | Exhibited inhibitory effects on *Porphyromonas gingivalis* | Enhanced the osseointegration | (Chen, et al. 2017) |
|  | N-FHA | Reduced bacterial expansion and biofilm development, exhibited enhanced cell adhesion and proliferation | Promoted osseointegration | (Wang, et al. 2014) |
|  | Se NPs | Exhibited significant inhibition of *Pseudomonas aeruginosa* | - | (Wang, et al. 2016) |
| Surface texturing | Cicada wing surface microstructure | Exhibited inhibition *Pseudomonas aeruginosa* | - | (Wang, et al. 2017) |
|  | Willow-like ZnO nanosheets | Demonstrated high efficacy against gram-positive bacterial | Demonstrated high efficacy against gram-positive bacterial | (Ye, et al. 2019) |
|  | Zn, oxygen plasma immersion ions, carbon fiber | Discovered to have excellent antibacterial properties against *Staphylococcus aureus* and *Staphylococcus epidermidis* | - | (Lu, et al. 2016) |
